# Changes in Hemodynamics and Tissue Oxygenation Saturation in the Brain and Skeletal Muscle Induced by Speech Therapy – A Near-Infrared Spectroscopy Study

**DOI:** 10.1100/tsw.2011.116

**Published:** 2011-06-09

**Authors:** U. Wolf, F. Scholkmann, R. Rosenberger, M. Wolf, M. Nelle

**Affiliations:** ^1^ Institute of Complementary Medicine KIKOM, University of Bern, Bern, Switzerland; ^2^ Biomedical Optics Research Laboratory, Division of Neonatology, Department of Obstetrics and Gynecology, University Hospital Zurich, Zurich, Switzerland; ^3^ Division of Neonatology, University Hospital Bern, Bern, Switzerland

**Keywords:** arts speech therapy, speech therapy, near-infrared spectroscopy, NIRS, physiology, brain research, hemodynamics, oxygenation, Mayer waves

## Abstract

Arts speech therapy (AST) is a therapeutic method within complementary medicine and has been practiced for decades for various medical conditions. It comprises listening and the recitation of different forms of speech exercises under the guidance of a licensed speech therapist. The aim of our study was to noninvasively investigate whether different types of recitation influence hemodynamics and oxygenation in the brain and skeletal leg muscle using near-infrared spectroscopy (NIRS). Seventeen healthy volunteers (eight men and nine women, mean age ± standard deviation 35.6 ± 12.7 years) were enrolled in the study. Each subject was measured three times on different days with the different types of recitation: hexameter, alliteration, and prose verse. Before, during, and after recitation, relative concentration changes of oxyhemoglobin (Δ[O_2_Hb]), deoxyhemoglobin (Δ[HHb]), total hemoglobin (Δ[tHb]), and tissue oxygenation saturation (StO_2_) were measured in the brain and skeletal leg muscle using a NIRS device. The study was performed with a randomized crossover design. Significant concentration changes were found during recitation of all verses, with mainly a decrease in Δ[O_2_Hb] and ΔStO_2_ in the brain, and an increase in Δ[O_2_Hb] and Δ[tHb] in the leg muscle during recitation. After the recitations, significant changes were mainly increases of Δ[HHb] and Δ[tHb] in the calf muscle. The Mayer wave spectral power (MWP) was also significantly affected, i.e., mainly the MWP of the Δ[O_2_Hb] and Δ[tHb] increased in the brain during recitation of hexameter and prose verse. The changes in MWP were also significantly different between hexameter and alliteration, and hexameter and prose. Possible physiological explanations for these changes are discussed. A probable reason is a different effect of recitations on the sympathetic nervous system. In conclusion, these changes show that AST has relevant effects on the hemodynamics and oxygenation of the brain and muscle.

### INTRODUCTION

Language is one of the most important abilities in man, and enables one to communicate and transmit even abstract content and meaning to others. Speech and language highly depend on intact brain functions. Language and speech processing have been investigated mostly with magnetic resonance imaging (MRI) and electroencephalography (EEG). Due to its advantages, near-infrared spectroscopy (NIRS) has been increasingly used in different studies to investigate brain physiology. So far, most of the research using NIRS has focused on neonates and small children, which included pitch, phonemic, and prosodic tasks, and recognition of the voice of the mother[1,2]. Speech and language are far less investigated in adults, with studies on verbal fluency, laterality, and language production[3,4,5], and are mainly in patients with psychiatric disorders[6,7,8,9]. There is also a lack of knowledge on the effects of speech therapy. Considering that speech therapy becomes increasingly important in both child development and rehabilitation, more research and knowledge about speech therapy is desirable. Two major fields in speech therapy are logopedic speech therapy and arts speech therapy (AST). While the first focuses on pronunciation and articulation[10], the latter is used to induce various processes in the patients, mainly related to respiration and blood circulation[11].

AST is a method within complementary medicine and has been practiced for decades for various medical conditions; it comprises listening and the recitation of different forms of speech exercises under the guidance of a licensed speech therapist. Exercises include declamation and recitation of prose texts and different types of poetic meters, such as hexameter and alliteration. The selection of the task depends on the patient's condition as well as the intended goal of the therapy. Applications of AST range from speech and language disorders (e.g., sigmatism, raucousness, stammer, and aphasia), disorders of the respiratory and cardiovascular system (e.g., bronchial asthma, arterial hypertension) and respiration in the broadest sense (e.g., sinusitis), and psychosomatic disorders/diseases (e.g., anxiety, depression). The achievements of AST are monitored by qualitative (e.g., general condition) as well as quantitative (e.g., blood pressure, respiration or heart rate, forced expiratory volume within 1 sec [FEV1]) parameters. Effects of AST have also been investigated by means of heart rate variability (HRV). It has been shown that AST provokes alterations in heart rate dynamics[12] and, in particular, reciting hexameter led to strong cardiorespiratory synchronization and a strengthening of vagus-related HRV parameters[13,14]. An easy understanding about the distinction of different poetic meters can be achieved by the comparison of hexameter and alliteration. While hexameter, the poetic meter of the *Iliad* and *Odyssey*, has a calming effect while listening to or reciting, alliteration, the poetic meter of, for example, the Norse *Edda*, induces alertness and acceleration of respiration and heart rate.

As there is indication that AST has clinical effects, and as it has been shown that speech and verbal tasks affect brain physiology, the aim of our study was to noninvasively investigate whether different types of speech, namely recitation of hexameter, alliteration, and prose, influence hemodynamics and oxygenation in the brain and skeletal leg muscle using NIRS.

### MATERIAL AND METHODS

#### Study Design

The study was carried out as a controlled, randomized (by computer) crossover trial.

#### Participants

Seventeen healthy volunteers (eight men and nine women, mean age ± standard deviation 35.6 ± 12.7 years) were enrolled in the study. All were without previous knowledge about speech therapy and their mother tongue corresponded to the one of the speech practices. Exclusion criteria were any impairment of lung function. Three participants were smokers with different cigarette consumptions (one participant: 40 pack years [total 292,000 cigarettes], two participants: <5 pack years [total 36,500 cigarettes]). The participants were asked to refrain from consuming any stimulants, such as caffeine, at least 2 h before the measurement.

Written informed consent was obtained prior to each measurement from all subjects. All participants were reimbursed for their travel expenses.

### Experimental Protocol

Each subject was measured three times on different days with the different types of speech practice: hexameter (repeating the first 12 verses from Goethe's "Achilleis"[15]), alliteration (repeating the first 11 verses of the "Thrymskvidha" from the Norse *Edda*[16]), and a prose text (the first part of "Der zweite Mann" written by Hugo Loetscher[17]). Since it was impossible to provide different speech exercises with exactly the same content, the texts were chosen in such that the quality of content was comparable. The hexameter and alliteration addressed issues of action and fighting, however in far distant times, and the prose text addressed action and struggle. The prose verse was chosen as a control condition because it includes speaking and that associated breathing pattern without expressing the poetic features of the other verses.

The sequence of the type of speech practice was randomized by computer. An arts speech therapist was present for each measurement. Before each measurement, the speech therapist shortly familiarized the subjects with the respective speech practice. During the measurement, the speech therapist recited the text, verse per verse, and left the subject enough time to repeat the verse. A verse corresponds to a line. All subjects were able to comfortably recite an entire line per breath. The prose text was recited in the same kind, i.e., text parts were repeated in one breath. Therefore, the length and effort spent on reciting individual parts of the different text forms can be considered as comparable. As is customary, the subjects repeated what they heard without having the texts in front of them. Each measurement included an 8-min baseline (spontaneous breathing), 10-min speech intervention, and a 20-min recovery. The subjects were comfortably seated in a chair with armrests and were asked to move as little as possible in order to avoid artifacts. During the measurements, the room illumination was dimmed to reduce interference with ambient light.

The protocol was approved by an Ethical Committee of the Canton of Zurich and was in accordance with the Declaration of Helsinki.

### Measurements

Relative concentration changes of oxyhemoglobin ($\Delta[O_2Hb]$ in μM), deoxyhemoglobin ($\Delta[HHb]$ in μM), and total hemoglobin ($\Delta[tHb]$ in μM), as well as the tissue oxygenation saturation ($StO_2$, i.e., $[O_2Hb]/[tHb]$ in %) were measured in the brain and in the skeletal leg muscle using a NIRS device (NIRO 300, Hamamatsu Photonics, Hamamatsu, Japan) during the whole duration of the experiment (38 min). The NIRS device is based on spatially resolved near-infrared reflectance spectroscopy using four wavelengths (778, 813, 850, and 913 nm) and described in detail elsewhere[18]. One NIRS sensor was attached over the left frontal lobe and at a location between $F_{p1}$ and $F_7$, according to the international 10/20 system[19]. The second NIRS sensor was attached vertically on the left calf muscle, with the upper edge at a 10-cm distance from the fibula's head.

While $\Delta[O_2Hb]$ and $\Delta[HHb]$ were calculated using the modified Lambert-Beer law[20], the calculation of $\Delta[tHb]$ and $StO_2$ (named THI and TOI by the manufacturer) was based on spatially resolved spectroscopy[18]. The Lambert-Beer law approach is sensitive to all the tissue penetrated by the light and thus also to superficial tissue. The spatially resolved spectroscopy approach cancels information from superficial tissue and is thus sensitive to deep layers of tissue[21], e.g., the brain. These two approaches enabled two different types of NIRS signals: those more sensitive to the superficial layers of the tissue (Δ[$O_2$Hb], Δ[HHb]) and those more sensitive to deep tissue layers (Δ[tHb], $StO_2$). The sensitivity differences with respect to different parts of the tissue were used as additional information to gain more insight into the physiological reactions caused by the recitation tasks.

Calculating Δ[tHb] requires assuming a light scattering coefficient of the measured tissue. Since light scattering may vary between tissues and individuals, there is a degree of uncertainty about the quantification of Δ[tHb]. However, this concerns only the size of the change and not its pattern or directions.

$StO_2$ quantifies the regional tissue oxygenation and is mainly related to changes in the arterial oxygen saturation and the end-tidal $CO_2$, and only weakly to changes in mean arterial blood pressure and cerebral blood volume[22]. The relative total hemoglobin concentration Δ[tHb] represents changes in the blood volume of the tissue and is related to blood flow[23].

### Data Analysis

Movement artifacts (MAs) in the recorded hemodynamic signals (Δ[$O_2$Hb], Δ[HHb], Δ[tHb], and $StO_2$) were removed using a novel method[24]. The method enables a semi-automatic detection and reduction of MAs.

For the data analysis, each hemodynamic signal was analyzed in the time and frequency domain. For both analyses, all data were divided into seven intervals ranging: (1) 3–5 min, (2) 5–10 min, (3) 10–15 min, (4) 15–20 min, (5) 20–25 min, (6) 25–30 min, and (7) 30–35 min. Thus, there was one preintervention interval (interval 1), two intervention intervals (intervals 2 and 3), and four postintervention intervals (intervals 4–7). The first 3 min were not considered in the data analysis.

For the analysis in the time domain, the median values were calculated for every interval.

The analysis in the frequency domain was performed by calculating the power spectra using Welch's method[25]. The power spectral density was determined in the interval from 0.02 to 0.05 Hz and 0.07 to 0.13 Hz. The latter corresponds to the frequency band of the Mayer waves, i.e., a low-frequency oscillation, which is present in the blood volume and oxygenation in humans[26,27,28]. Minimal effects were seen in the band from 0.02 to 0.05 Hz; hence, are not shown in the results.

Data are shown relative to the first baseline period. A nonparametric test (paired Wilcoxon test) was applied to investigate (1) whether the recitation causes changes in the median values and/or in the Mayer wave power (MWP) of the hemodynamic signals, and (2) whether these changes are different between the three recitation forms. A significant change ($p < 0.05$) occurred with the recitation when the change was significant in at least one of the two intervention intervals (2 and 3). The cross-sectional comparisons were calculated for each interval.

## RESULTS

### Changes in Hemodynamics and Tissue Oxygenation in the Brain

#### *Analysis of the Median Values*

The following statistically significant hemodynamic and oxygenation changes (all with respect to baseline) were found (subfigures a–c in Fig. 1):

1. During the speech intervention, a decrease in Δ[$O_2$Hb] and $StO_2$ compared to baseline was observed in all three types of recitation (hexameter, alliteration, and prose).
2. During the recovery phase, Δ[$O_2$Hb] increased and Δ[HHb] decreased for alliteration; Δ[tHb] increased in the recovery phase in all three types of recitation, but not significantly.

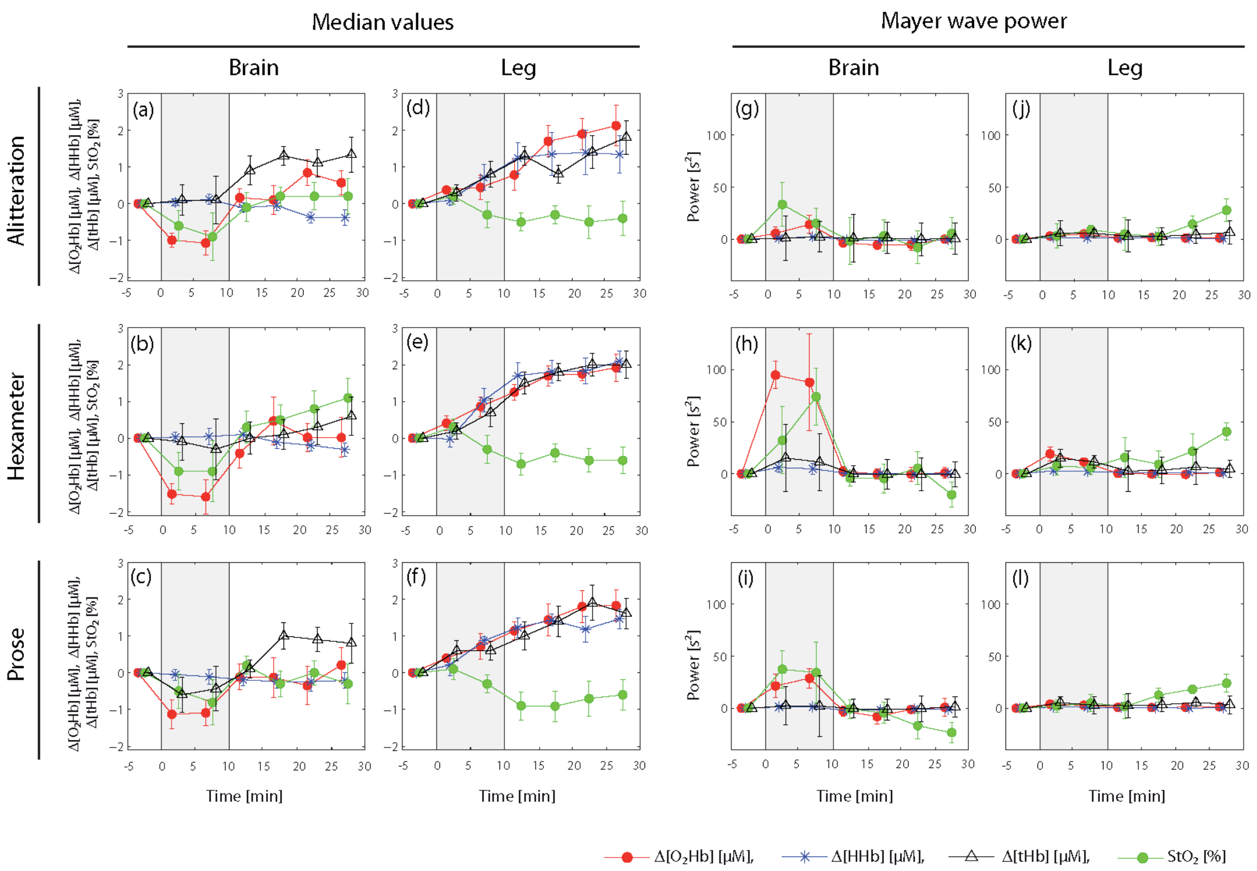

**FIGURE 1.** Results of the data analysis in the time and frequency domain. Subfigures a–f show the changes in the hemodynamic and oxygenation signals during the three different speech tasks measured in the brain (a–c) and the leg (d–f). For the brain, clear changes are visible during recitation, mostly a reduction in $\Delta[O_2Hb]$ and $StO_2$, which is probably due to a $CO_2$ reaction and an increase in oxygen consumption. In the leg, $\Delta[O_2Hb]$, $\Delta[HHb]$, and $\Delta[tHb]$ increase continuously, probably due to venous pooling. The corresponding changes in the MWP are shown in the subfigures g–i and j–l, respectively. In the brain, MWP is elevated for $\Delta[O_2Hb]$ and $StO_2$ during recitation, with a significantly higher increase during hexameter recitation. This probably indicates an activation of the sympathetic nervous system. The data were set to zero during the baseline period before recitation to enhance visibility of the changes. The error bars represent the bootstrap estimate of the standard error of the median, calculated according the approach presented in Martinez and Martinez[47].

3. When comparing the three types of recitation, significant differences were only found in the postintervention periods, i.e., $StO_2$ was significantly higher for hexameter and alliteration compared to prose.

### *Analysis of the MWP*

The following significant changes (all with respect to baseline) in the MWP were found (subfigures g–i in Fig. 1):

1. During hexameter and prose recitation, MWP increased for $\Delta[O_2Hb]$ and $\Delta[tHb]$, while the increase during alliteration was not significant. During hexameter and alliteration recitation, MWP increased significantly for $\Delta[HHb]$ and $StO_2$.

2. During the recovery phase, after reciting prose, there was a significant decrease in MWP of Δ[$O_2$Hb], Δ[HHb], and Δ[tHb] compared to baseline, while alliteration and hexameter showed no significant changes during recovery.
3. When comparing the three types of recitation, for the MWP of Δ[$O_2$Hb] and Δ[tHb], the MWP increase was significantly larger for the recitation of the hexameter verse compared to alliterative and prose verse, while for the MWP of Δ[HHb] and $StO_2$, the increase during hexameter recitation was significantly larger compared to prose only.

### Changes in Hemodynamics and Tissue Oxygenation Saturation of the Skeletal Muscle

#### *Analysis of the Median Values*

The following statistically significant hemodynamic and oxygenation changes were found (subfigures d–f in Fig. 1):

1. During the speech intervention, an increase in Δ[$O_2$Hb] and Δ[tHb] compared to baseline was observed in all three types of recitation, while Δ[HHb] only increased for alliteration and hexameter.
2. During the recovery phase, Δ[tHb] and Δ[HHb] increased significantly for all three types of recitation. Δ[$O_2$Hb] significantly increased only during alliteration. $StO_2$ decreased in all three types of speech practice in the recovery phase, but not significantly for the prose recitation.
3. When comparing the three types of recitation, no significant differences were found.

#### *Analysis of the MWP*

The following statistically significant changes in MWP were found (subfigures j–l in Fig. 1):

1. During the speech intervention, an increase in MWP for Δ[$O_2$Hb] was found for the hexameter and prose verse, the MWP for Δ[HHb] and Δ[tHb] increased for all three types of recitation, and the MWP of $StO_2$ increased only during the alliteration.
2. During the recovery phase, the MWP of Δ[$O_2$Hb] increased after prose recitation, the MWP of Δ[HHb] increased after hexameter and alliteration, and the MWP of Δ[tHb] and $StO_2$ increased for all three types of recitation.
3. When comparing the three types of recitation, the increase in MWP of Δ[$O_2$Hb] was significantly larger for the recitation of the hexameter verse compared to alliterative and prose verse; the increase in MWP of Δ[tHb] was significantly higher for hexameter and alliteration verse compared to prose.

## DISCUSSION

### Changes in Hemodynamics and Tissue Oxygenation Saturation in the Brain and Skeletal Muscle

#### *Changes in Median Values*

Cerebral Δ[tHb] did not significantly change during the recitation of any verse. Since Δ[tHb] is a measure of deep tissue and is also correlated with cerebral blood flow (CBF) as explained in the Methods section, this indicates that CBF did not change. The decrease in cerebral Δ[$O_2$Hb] and $StO_2$ during the speech exercise for all three types of recitation consequently indicates an increase in oxygen consumption. In the course of brain activation by recitation, normally an increase in CBF and Δ[$O_2$Hb] is expected, rather than no change or a decrease. This standard pattern has been observed in many functional brain studies (e.g., [5,29]) and is explained by an increase in oxygen consumption in the activated brain area, which triggers a larger increase in CBF, so-called neurovascular coupling, which causes an increase in Δ[tHb] (blood vessels open up to increase CBF)[23]. The increase in CBF is so large that it overcompensates for the increase in oxygen consumption and a net increase in Δ[$O_2$Hb] is observed[29]. However, reciting, by way of an altered respiration, may have an influence on the $CO_2$ concentration in blood. It is well known that accelerated breathing leads to a decrease in $CO_2$ blood level, which in turn induces constriction of the blood vessels in the brain and thereby results in a decreased blood flow and volume[30]. Recitation follows a rhythm where one phrase is spoken per breath. Although this decreases the breathing rate, it increases the volume of each breath, i.e., more $CO_2$ is removed and consequently may very well have a decreasing effect on the $CO_2$ level in the blood.

An increase in systemic blood pressure may also have occurred due to an increase in sympathetic activation. This has been shown to affect Δ[$O_2$Hb] and Δ[HHb][31], i.e., the superficial variables. It is less likely to affect the Δ[tHb] and $StO_2$ because the variables are not sensitive to superficial tissue and the autoregulation of CBF keeps CBF independent of blood pressure changes. Thus, although an effect of changes in blood pressure cannot be excluded, in our opinion, the $CO_2$ is likely to be the most important systemic influence.

The observed effects could thus be explained by an activation of the brain, which leads to an increased oxygen consumption. Neurovascular coupling strives to increase the CBF to increase the oxygen supply, while the decrease in $CO_2$ counteracts and, consequently, the CBF and Δ[tHb] remain more or less stable. Due to the unchanged CBF, the increase in oxygen consumption leads to a decrease in Δ[$O_2$Hb] and $StO_2$.

The $CO_2$ reaction may mask other potential differences between hexameter and alliteration. An approach to investigate potential differences between hexameter, alliteration, and prose, avoiding the $CO_2$ reaction would be to only allow the subjects to listen to the recitation of the AST therapist or to follow the recitation with “inner speech”, i.e., without actually speaking aloud and thereby avoiding a significant alteration in breathing. This reasoning is based on the mirror neurons hypothesis[32], i.e., that a subject listening to AST is actually activating the same areas of the brain as the person reciting. Whether these types of silent recitation are sufficient to elicit measurable signals is unclear. It is, however, known from functional studies of the motor cortex that imagining a movement induces significant changes[33]. Also, when not actively speaking, but only listening to recitation, the breathing pattern may be changed, although probably to a much lesser extent.

After the recitation, Δ[tHb] increases, although not significantly, which may indicate a recovery overshoot.

In the leg, the continuous increase in Δ[$O_2$Hb], Δ[HHb], and Δ[tHb] can be explained by an accumulation of blood and reduced venous drainage in the calf muscle during the measurement due to a sedentary position of the subject. This is enhanced by sitting still, i.e., reducing the activity of the muscle pump. The hypothesis of venous pooling is also supported by the decreased $StO_2$. In summary, the predominant effect in the leg is a continuous accumulation of venous blood. One option to avoid this effect in the future would be to have the subjects seated with their legs elevated or to have them in a supine position.

#### *Changes in MWP*

The increases in the MWP in cerebral Δ[$O_2$Hb] and Δ[tHb] during all three types of recitation (although not significant for alliteration) can be explained by a response of the autonomic nervous system to the recitation tasks. In particular, studies showed that Mayer waves are tightly related to the sympathetic vasomotor tone, e.g., the blockade of the α-adrenoreceptor (which mediates a sympathetic response) causes a strong attenuation of the MWP[34,35,36,37,38]. An increase in MWP was found in humans under mental stress[39]. While this is true for healthy people, this association is disturbed in patients with congestive heart failure, where the MWP is decreased in rest and during sympathoexcitatory maneuvers (e.g. tilt, physical exercise, and mental stress) compared to healthy people[40,41,42,43,44]. The observation that the Mayer waves are most pronounced in the cerebral $\Delta[O_2Hb]$ and $\Delta[tHb]$ signals is in agreement with previous literature[28]. The increases in MWP could also be initiated by systemic changes since experiments showed that MWP $\Delta[O_2Hb]$ and $\Delta[HHb]$ changed synchronously with blood pressure and the heart rate[45,46].

The most pronounced and significant increase of the MWP during hexameter recitation indicates a particularly strong activation of the sympathetic nervous system. Additionally, the MWP during recitation of the hexameter is significantly higher than that for alliteration or prose. This indicates that the different types of recitation indeed affect the sympathetic nervous system differently. Beside the form of the speech exercises, the content may play a role. Due to the impossibility of providing different speech exercises with exactly the same content, texts with similar quality of content were chosen. All speech exercises referred to action, fighting, and struggle. Additionally, the subjects, when asked about the content of the speech exercises (1) could barely remember the content and (2) did not describe specific differences between the content. Therefore, it is most likely that the observed changes relate to the form of verses of the speech exercises. One option for future studies would be to transform the content of the hexameter and alliteration into prose.

The finding of the increase in MWP in muscle $\Delta[tHb]$ and $\Delta[HHb]$ in the recovery phases of all three types has not been described before. It might be that the accumulation of blood in the leg muscle leads to a higher susceptibility to Mayer waves in the arterial blood vessels.

### CONCLUSION

In conclusion, significant changes in hemodynamics and oxygenation were observed during the measurements for the three different types of recitation.

In the brain, the most pronounced effect was a decrease in $\Delta[O_2Hb]$ and $StO_2$ during recitation due to increased oxygen consumption, while, interestingly, $\Delta[tHb]$ and, consequently, CBF remained stable. There was a rebound effect for CBF during recovery for alliteration. Because the $CO_2$ reaction is so predominant, it may mask potential differences in hemodynamics and oxygenation between hexameter and alliteration. However, in a protocol where the subjects are actively reciting, the $CO_2$ reaction can hardly be avoided.

In the leg, the predominant effect was venous pooling and a concurrent decrease in $StO_2$ due to the sedentary posture of the subjects.

The impact of different recitation types on the MWP is another finding of our study. The strong and significant increase in MWP during the recitation of the hexameter shows that (1) the type of recitation influences the MWP and (2) in particular, the hexameter recitation is a method to increase the activity of the sympathetic nervous system, which could be used in a therapeutic context of AST.

For future studies, it would be relevant to (1) measure breathing, heart rate, blood pressure, and the $CO_2$ concentration for the different types of recitation; (2) measure cerebral hemodynamics and oxygenation; and (3) test whether the recitation with "inner speech" has an effect on cerebral hemodynamic and oxygenation.

### ACKNOWLEDGMENT

We thank all subjects and the arts speech therapists for their participation in this study.
